# Assessing therapeutic change in patients with severe dissociative disorders: the progress in treatment questionnaire, therapist and patient measures

**DOI:** 10.1080/20008198.2017.1380471

**Published:** 2017-10-13

**Authors:** Hugo Schielke, Bethany Brand, Angelika Marsic

**Affiliations:** ^a^ Psychology Department, California Department of State Hospitals, Napa, CA, USA; ^b^ Psychology Department, Towson University, Towson, MD, USA; ^c^ Psychology Department, California Department of Corrections and Rehabilitation, California Correctional Institution, CA, Tehachapi, USA

**Keywords:** Dissociative identity disorder (DID), dissociative disorders (DD), posttraumatic stress disorder (PTSD), PTSD, dissociative subtype, complex trauma, progress, outcome, assessment, questionnaires, trastorno de identidad disociativo (TID), trastornos disociativos (TD), trastorno de estrés postraumático (TEPT), TEPT, subtipo disociativo, trauma complejo, progreso, resultado, evaluación, cuestionarios, 分离身份障碍（DID）, 分离障碍（DD）, 创伤后应激障碍（PTSD）, PTSD分离亚型, 复杂创伤, 进展, 结果, 评估, 问卷

## Abstract

**Background**: Treatment research for dissociative identity disorder (DID) and closely related severe dissociative disorders (DD) is rare, and has been made more difficult by the lack of a reliable, valid measure for assessing treatment progress in these populations.

**Objective**: This paper presents psychometric data for therapist and patient report measures developed to evaluate therapeutic progress and outcomes for individuals with DID and other DD: the Progress in Treatment Questionnaire – Therapist (PITQ-t; a therapist report measure) and the Progress in Treatment Questionnaire – Patient (PITQ-p; a patient self-report measure).

**Method**: We examined the data of 177 patient–therapist pairs (total *N *= 354) participating in the TOP DD Network Study, an online psychoeducation programme aimed at helping patients with DD establish safety, regulate emotions, and manage dissociative and posttraumatic symptoms.

**Results**: The PITQ-t and PITQ-p demonstrated good internal consistency and evidence of moderate convergent validity in relation to established measures of emotional dysregulation, dissociation, posttraumatic stress disorder, and psychological quality of life, which are characteristic difficulties for DD patients. The measures also demonstrated significant relationships in the hypothesized directions with positive emotions, social relations, and self-harm and dangerous behaviours. The patient-completed PITQ-p, which may be used as an ongoing assessment measure to guide treatment planning, demonstrated evidence of stronger relationships with established symptom measures than the PITQ-t.

**Conclusions**: The PITQ-t and PITQ-p merit use, additional research, and refinement in relation to the assessment of therapeutic progress with patients with DD.

## Abstract (es)

**Planteamiento**: La investigación del tratamiento para el trastorno de identidad disociativo (TID) y los trastornos disociativos (TD) graves que están estrechamente relacionados es poco frecuente y se ha dificultado por la falta de una medida fiable y válida para evaluar el progreso del tratamiento en estas poblaciones.

**Objetivo**: Este artículo presenta los datos psicométricos de las medidas de informes de terapeutas y pacientes desarrolladas para evaluar el progreso terapéutico y los resultados en personas con TID y otros TD: El Cuestionario del progreso del tratamiento - terapeuta (PITQ-t, siglas en inglés de Progress in Treatment Questionnaire - Therapist; una medición mediante informe del terapeuta) y el Cuestionario del progreso del tratamiento - paciente (PITQ-p, siglas en inglés de Progress in Treatment Questionnaire - Patient; una medición mediante autoinforme del paciente).

**Método**: Se examinaron los datos de 177 pares de pacientes-terapeutas (N total = 354) que participaron en el TOP DD Network Study, un programa de psicoeducación online destinado a ayudar a los pacientes con TD a establecer seguridad, regular emociones y manejar síntomas disociativos y postraumáticos.

**Resultados**: El PITQ-t y PITQ-p demostraron buena consistencia interna y evidencia de una validez convergente moderada en relación a las medidas establecidas de desregulación emocional, disociación, trastorno por estrés postraumático y calidad de vida psicológica - dificultades características en pacientes con TD. Las medidas también demostraron relaciones significativas en el sentido establecido por la hipótesis en cuanto a emociones positivas, relaciones sociales, y conductas autolesivas y peligrosas. El PITQ-p completado por el paciente, que puede usarse como una medida de evaluación continua para guiar la planificación del tratamiento, evidenció relaciones más fuertes con las medidas establecidas de los síntomas que el PITQ-t.

**Conclusiones**: El PITQ-t y PITQ-p justifican su uso, investigación adicional y refinamiento en relación con la evaluación del progreso terapéutico con pacientes con TD.

## Abstract (zh)

**背景:**对分离身份障碍（DID）和与其紧密相关的分离障碍（DD）的治疗研究较少，缺少可靠有效的测量使评估这个群体的治疗进展更加困难。

**目标**：本文呈现了治疗师报告和病人自我报告的心理测量数据用于评估DID和DD病人的治疗进展和结果：治疗进展问卷-医师版（PITO-t，治疗师报告）和治疗进展问卷-患者版（PITO-p，病人自我报告）。

**方法**：我们分析了177对病人-医生组合参与TOP DD网络研究（一个帮助DD病人建立安全感、调控情绪和管理分离和创伤症状的网上心理教育项目）的数据（总体n=354）。

**结果**：PITQ-t 和 PITQ-p显示了良好的内部一致性，与情绪失调、分离、创伤后应激障碍和心理生活质量-DD病人的特征性困难表现出中等的聚合效度。和假设一致，这个测量也表现出积极情绪、社会关系、自我伤害和危险行为的显著相关。病人完成的PITQ-p，可以用作持续的评估测量来指导治疗计划，与已成熟的测量工具之间比PITQ-t表现出更强的关联。

**结论**：PITQ-t 和 PITQ-p和DD病人的治疗进展评估的关系说明它们是值得使用、可以用于研究中和进行改进的的工具。

Epidemiological studies suggest that dissociative identity disorder (DID) occurs in approximately 1.5% of the general U.S. population (American Psychiatric Association [APA], 2013; Johnson, Cohena, Kasena, & Brook, 2006) and 1% of all available community samples internationally, and that prevalence increases with level of care (median outpatient prevalence rate: 2.5%, median inpatient prevalence rate: 5%; Foote, 2016). Dissociative disorder not otherwise specified (DDNOS), the DSM-IV-TR diagnosis assigned to patients with symptoms similar to DID (Spiegel et al., 2011), is more than twice as prevalent in the U.S. (4.4%; Johnson et al., 2006). These severe dissociative disorder (DD) patients frequently present with myriad difficulties, including emotion dysregulation, intrusions of traumatic experiences and other symptoms of posttraumatic stress disorder (PTSD), dissociation, self-harm, suicidality, attachment and relationship difficulties (including mistrust of therapists), and poor quality of life (Brand, Classen, Lanius et al., 2009; Foote, Smolin, Kaplan, Legatt, & Lipschitz, 2006; Johnson et al., 2006; Liotti, 1992; Mueller-Pfeiffer et al., 2012; Pasquini, Liotti, Mazzotti, Fassone, & Picardi, 2002). Treatment research for DD patients is scarce, however (Brand, Classen, McNary, & Zaveri, 2009), and is made more difficult by the lack of a reliable, validated measure for assessing DD treatment progress. To address this need, this paper presents psychometric data for two brief measures for assessing DD treatment progress and outcomes: the Progress in Treatment Questionnaire – Therapist (PITQ-t; a therapist report instrument), and the Progress in Treatment Questionnaire – Patient (PITQ-p; a patient self-report instrument). These measures represent new versions of the Progress in Treatment Questionnaire (PITQ; Brand, Classen, Lanius et al., 2009), a therapist report measure designed to assess DD patient capacities targeted in the treatment of adult DID patients.

## 1.1. The Progress in Treatment Questionnaire

The Progress in Treatment Questionnaire (PITQ) was developed for the naturalistic Treatment of Patients with Dissociative Disorders (TOP DD) study (Brand, Classen, Lanius et al., 2009, Brand et al., 2013). The TOP DD study was a 30-month naturalistic study of DD patients’ progress in individual therapy. Prior to this study, only one DID-specific progress/outcome measure had been developed: the Dimensions of Therapeutic Movement Instrument (DTMI; Kluft, 1994). The DTMI is completed by clinicians who rate their DD patients in 12 areas, including the therapeutic relationship, self-regulation, need for external support, phenomena related to dissociative self-states (DSS),^1^ interpersonal functioning, and sense of well-being. The DTMI offers promise as a therapist-completed assessment measure, but has not been systematically assessed for reliability or validity (Choe & Kluft, 1995; Kluft, 1994) and is sufficiently complex that it was deemed too difficult to use in online research such as the TOP DD study.

For these reasons, the TOP DD investigators developed the PITQ. As described in Brand, Classen, Lanius et al. (2009), the DD treatment experts involved in the study designed the therapist-completed PITQ by consensus to assess DD patients’ ability to safely and effectively manage their emotions, symptoms, and relationships; these are adaptive capacities developed across the three stages of treatment described in the International Society for the Study of Trauma & Dissociation’s (ISSTD) *Guidelines for Treating Dissociative Identity Disorder in Adults* (2005, 2011). PITQ item development was informed by the ISSTD Treatment Guidelines, the items in the DTMI, and the TOP DD team’s extensive clinical experience. PITQ items most closely linked with the first stage of treatment, *establishing patient safety and stabilization* (Brand et al., 2012; ISSTD, 2011), query patients’ ability to maintain safety (i.e. not engage in behaviours that put their life or health at risk, including non-suicidal self-injury, suicidality, and other dangerous behaviours); effectively collaborate with their therapist; understand and manage DD- and PTSD-related symptoms; manage self-care/daily living; view themselves and others in realistic ways that are free from trauma-based distortions; maintain healthy personal and professional relationships; tolerate, regulate, and identify affect; and develop cooperation with DSS. PITQ items most closely linked with the second phase of treatment, *processing trauma*, query patients’ ability to process trauma and identify and understand the roles of DSS. PITQ items most closely linked with the third phase of treatment, *reconnection*, query patients’ ability to resolve (Kluft, 1993; Kluft & Loewenstein, 2007) the conflicts between and/or integrate DSS and find ways to make life feel meaningful and rewarding. An item also queries patients’ ability to pleasurably experience sexual intimacy, which can be difficult for individuals with DDs. The PITQ’s 29 items ask therapists to indicate what percentage of the time their patient has demonstrated the queried abilities over the prior six months. Each item offers 11 response options ranging from 0% (never) to 100% (always) in 10% intervals (i.e. 0%, 10%, 20%, etc.).

### 1.1.1. Psychometric properties of the PITQ

#### 1.1.1.1. Reliability

The PITQ has demonstrated evidence of internal consistency (i.e. internal reliability) (Brand, Classen, Lanius et al., 2009, Brand et al., 2013; Marsic, Brand, Schielke, & Putnam, 2013) and test-retest reliability (Marsic et al., 2013) in studies with DD patients. In a 30-month prospective, naturalistic study of the treatment of DD patients, the PITQ demonstrated Cronbach’s coefficients ranging from .94 to .95 (see Brand, Classen, Lanius et al., 2009, and Brand et al., 2013 for methodology details). In an IRB-approved study aimed at assessing the test-retest reliability of the PITQ, we invited therapists who had participated in the naturalistic study to complete the PITQ at two time points (two weeks apart) for a single adult outpatient diagnosed DID or DDNOS by DSM-IV-TR criteria (Marsic et al., 2013). To increase generalizability, we did not exclude patients based on psychiatric comorbidity. No patient-identifying information was collected, and therapist participants’ data was de-identified. Participation was excluded for therapists without access to the internet or unable to read English. A total of 117 therapists consented to participate in the study; 74 (53 females and 21 males) completed the PITQ at both Time 1 and Time 2 (retention rate: 63%). Responses were matched by participant code numbers. Analysis of participants’ test responses revealed a strong Pearson’s product moment correlation (.87, *p* > .01) between the two administrations, suggesting good test-retest reliability and temporal stability over a two-week period (*M* = 16.6 days). The probability of these findings occurring in the same population by chance was found to be <1% [*t*(73) = −2.72, *p* = .008], and a Pittman-Morgan test of homogeneity of variance indicated that there was no significant difference in variance between the two samples [*t*(72) = .61, *p* = .54] (*SD* Time 1 = 16.09, *SD* Time 2 = 14.99). The PITQ demonstrated good internal consistency at both time points (Cronbach’s alpha: .95 and .94, respectively).

#### 1.1.1.2. Validity

Abilities assessed by the PITQ correspond with DD treatment experts’ endorsed intervention targets (see Table 1). As a measure intended to assess patients’ development of adaptive capacities in managing safety, emotion, symptoms, and relationships, PITQ scores should be higher in patients in later stages of treatment. Consistent with this theory, in cross-sectional comparisons, mean PITQ scores were significantly higher in patients in the ‘processing trauma’ and ‘reconnection’ stages than in patients in stage 1 (*p* < .01; Hedge’s *g*, ‘processing’ vs. ‘establishing safety’: 1.73; Hedge’s *g*, ‘reconnection’ vs. ‘establishing safety’: 2.90; Brand et al., 2009). Similarly, in longitudinal analyses of TOP DD participants over 30 months, PITQ scores increased over time (Brand et al., 2013); these increases in PITQ scores coincided with reductions in symptoms of dissociation, depression, general distress, and PTSD, as well as with reductions in substance use and dangerous behaviour.

**Table 1.** Relationships between abilities assessed by PITQ-t, PITQ-p, and expert intervention targets.

Interventions that experts endorse using‘very often’ during at least one stage of treatment^a^	PITQ-t items assessing abilitytargeted by intervention^b^	PITQ-p items assessing abilitytargeted by intervention^c^
Establishing safety	1, 2	12, 18, 19
Establishing/repairing alliance	5	2
Teaching/practicing grounding	8	7, 28
Educating about disorders/treatment	4	1
Diagnosing psychiatric illnesses	*	*
Teaching/practicing self-care	14	14
Developing healthy relationships	17	17
Affect tolerance and impulse control	11, 12	10, 11
Stabilizing from current stressors	6	4, 5
Teaching/practicing containment	7	6
Ego strengthening activities	3, 20	22, 24
Acceptance of DD diagnosis	4	1
Processing when and why dissociation occurs	15, 16	15, 16
Assess response to medications	*	*
CBT focused on cognitions	3, 9, 13, 20, 21, 24	3, 8, 13, 22, 23, 24, 27, 28
Awareness of emotion	10	9
Processing patient’s reactions to therapy	5	2
Stabilizing following intrusions from alleged perpetrators	6	4, 5
Cooperation with/between parts	26, 27	29, 32
Teaching/discussing attachment	^ (17, 20, 21)	^ (17, 22, 23)
Identifying/working with parts	25	30
Discussing therapeutic relationship	5	2
Awareness of body sensation	10	9
Exposure to traumatic memories/abreaction	18, 23	20, 26

^a^ Interventions endorsed by at least 45% of experts; Brand et al. (2012).

^b^ PITQ-t items 4–29 (see Table 5 for item content) are taken verbatim from PITQ items; PITQ-t items 1–3 rephrase the content of the remaining 3 PITQ items for clarity.

^c^ See Table 7 for PITQ-p item content.

* Not a patient-targeted ability.

^ PITQ family of measures does not explicitly query, but is very closely related to (items in parentheses).

**Table 2.** Therapist characteristics.

	%	(*n*)	*M*	*SD*	Min	Max
***Gender***						
Female	83	(147)				
Male	17	(30)				
***Primary Therapist Orientation***						
Cognitive-Behavioural	12	(22)				
Psychodynamic	44	(77)				
Family Systems	5	(9)				
Humanistic/Experiential	14	(24)				
Other	25	(45)				
***Treatment Settings^a^***						
Private Practice	75	(133)				
Clinic/Hospital Outpatient	31	(55)				
Hospital Inpatient/Partial	4	(7)				
School	2	(3)				
Forensic	1	(2)				
Other	7	(13)				
***Therapist Experience***						
Years in Practice		(177)	17.75	9.20	1	44
Years Treating DD		(177)	10.63	7.78	1	34
Years Treating Trauma		(177)	15.48	8.64	1	44

^a^ Multiple settings could be indicated.

**Table 3.** Patient characteristics.

	%	(*n*)	*M*	*SD*	Min	Max
***Gender***						
Female	89	(157)				
Male	11	(19)				
Transgender	.6	(1)				
***Age***			41.42	10.75	19	68
***Race/Ethnicity***						
Caucasian	84	(148)				
Black	3	(6)				
Latino/Hispanic	5	(8)				
Asian	.6	(1)				
Other	8	(14)				
***Therapist-Reported Patient Abuse History***						
Was the patient neglected as a child?	67	(118)				
Was the patient emotionally or psychologically abused as a child?	86	(153)				
Was the patient physically abused as a child?	67	(116)				
Did the patient witness domestic violence as a child?	41	(73)				
Was the patient sexually abused as a child?	83	(147)				
***Therapist-Assigned DD Diagnosis***						
Dissociative Identity Disorder	75	(133)				
Dissociative Disorder Not Otherwise Specified (DSM-IV-TR)	23	(41)				
Other Specified DD (DSM 5)	2	(3)

**Table 4.** Descriptive statistics.

	*M*	*SD*	Min	Max
***Measures***				
PITQ-t	48.86	12.39	14.83	82.07
PITQ-p	44.25	14.67	11.56	80.63
PCL-C	61.37	11.19	29.00	85.00
DES	42.00	20.08	3.21	86.07
DERS	119.41	21.53	61.00	171.00
WHOQOL-BREF Psychological Domain	8.77	2.47	4.00	16.00
WHOQOL-BREF Social Domain	10.33	3.20	4.00	17.33
***Other variables***				
Self-harm^a^	3.06	5.56	0	30
Dangerous behaviour ^b^	1.03	3.03	0	30
Impulsive^c^	4.11	6.52	0	30
Positive emotions^d^	12.86	9.45	0	30

^a^ ‘On how many of the past 30 days did you purposefully hurt yourself (for example, cut yourself)?’

^b^ ‘How many times in the past 30 days have you done something that in retrospect was dangerous enough to kill you?’

^c^ ‘On how many of the past 30 days did you do something very impulsive (spending sprees, lost your temper and really shouted at someone, threatened to or actually harmed someone else, driven far too fast, done anything against the law, etc.)?’

^d^ ‘On how many of the past 30 days did you feel some good feelings even if it was for a brief period (e.g. happiness, contentment, joy)?’

**Table 5.** PITQ-t item-total statistics.

	Corrected Item-Total Correlation	Cronbach’s Alpha if Deleted
1. (R) Engages in self-injurious behaviour (e.g. cutting, burning) or suicide attempts	.37	.91
2. (R) Engages in potentially self-damaging acts such as abusing substances, purging, shoplifting, driving unsafely	.24	.91
3. (R) Identity is strongly tied to being a victim of abuse	.09	.92
4. Understands that they have a dissociative disorder (DD) and generally acknowledges that this diagnosis is accurate	.40	.91
5. Able to maintain a strong treatment alliance and, when there are disruptions to the alliance, able to work productively to repair it	.46	.91
6. Knows and uses self-soothing strategies (e.g. any type of calming strategy that is not used explicitly to contain PTSD symptoms or prevent dissociation) when they are needed	.56	.90
7. Knows and uses containment strategies (e.g. hypnotic or imagery techniques used to contain intrusive PTSD symptoms) when they are needed	.54	.90
8. Knows and uses grounding techniques to prevent self from going numb, zoning out, having amnestic lapses when they are needed (e.g. techniques such as muscle contractions, movement, or touching an object to avoid dissociating)	.47	.91
9. Keeps oriented in the present (i.e. does NOT get confused about past and present)	.46	.91
10. Shows good awareness of his/her emotions and feels his/her body sensations	.68	.90
11. Shows good affect tolerance (can feel emotions without getting overwhelmed)	.69	.90
12. Shows good impulse control (e.g. can feel angry or depressed without acting it out)	.50	.91
13. Is aware that the trauma was not his/her fault	.50	.91
14. Manages daily functioning well (e.g. managing hygiene, maintaining a home, paying bills)	.37	.91
15. Has continuous awareness of behaviours, that is, the patient does not report time loss or other signs of amnesia (e.g. no behaviours done out of their awareness, no possessions for which they can’t recall how they obtained them, etc.)	.55	.90
16. Able to deal with stressful situations without dissociating	.61	.90
17. Able to maintain healthy personal and professional relationships with other people	.61	.90
18. Able to experience grief stemming from trauma-related losses	.56	.90
19. Has found ways to make life feel meaningful and rewarding	.66	.90
20. Has a generally positive view of him/herself	.69	.90
21. Has a generally positive view of other people	.56	.90
22. Able to experience sexual intimacy without difficulties such as intense shame, flashbacks or dissociation and with some pleasure	.23	.91
23. Able to tolerate doing trauma focused abreactive work (i.e. able to express intense affect about past trauma, talk in detail about traumatic events, as well as explore the meaning, impact, and conflicts related to trauma)	.52	.91
24. Has awareness that all dissociated self-states are part of himself/herself and share one body (i.e. does not believe one alter can ‘kill’ another and survive the suicide)	.41	.91
25. Knows parts and understands their functions (i.e. what purposes they serve, such as helping manage feelings related to trauma)	.56	.90
26. Shows good internal communication and cooperation among parts	.63	.90
27. Has reliable co-consciousness with all parts	.58	.90
28. Has integrated at least two parts/self-states	.33	.91
29. Has integrated all parts and no longer experiences amnesia, voices, passive influence or other signs of identity fragmentation	.37	.91

(R) = reverse scored.

**Table 6.** PITQ-t and PITQ-p correlations with measures and variables of interest and significance of comparative strength of PITQ-t and PITQ-p correlations with measures and variables of interest.

	PITQ-t	PITQ-p	*r*_tp_	*t*	df	*p*	99% CI
**Measures**							
PCL-C	−.41**	−.47**	.50**	.81	174	.42	−.12–.22
DES	−.29**	−.42**	.50**	1.97	174	.05	−.04–.31
DERS	−.35**	−.67**	.50**	5.51	174	<.001**	.16–.48
WHOQOL-BREF Psychological	.45**	.64**	.50**	−3.21	174	<.001**	−.35–-.04
WHOQOL-BREF Social	.22**	.28**	.50**	−.76	174	.45	−.24–.13
**Other variables**							
Self-harm^a^	−.37**	−.34**	.50**	−.49	174	.62	−.21–.14
Dangerous behaviour^b^	−.31**	−.20**	.50**	−1.40	174	.16	−.29–.09
Impulsive^c^	−.23**	−.21**	.50**	−.22	174	.83	−.20–.17
Positive emotions^d^	.21**	.31**	.50**	−1.39	174	.17	−.28–.09

^a–d^: See Table 4.

* *p *< .05 (2-tailed), ** p < .01 (2-tailed), *r*_tp_ = correlation between PITQ-t and PITQ-p.

**Table 7.** PITQ-p item-total statistics.

	Corrected Item-Total Correlation	Cronbach’s Alpha if Deleted
1. I have been diagnosed with a dissociative disorder and agree that this diagnosis is correct	.17	.92
2. I collaborate well with my therapist and, when there are problems between us, I talk to my therapist about them so that we can resolve them together	.36	.92
3. I am compassionate and fair with myself, that is, I respond to myself with as much empathy as I would show someone else in the same situation	.52	.92
4. I’m aware of the thoughts, feelings, and body sensations that indicate I’m getting anxious or overwhelmed	.44	.92
5. I use relaxation techniques (such as relaxation exercises, safe place imagery, music) to safely help myself relax and feel better when I begin to get anxious or overwhelmed	.54	.92
6. I manage intrusive memories and flashbacks using containment strategies (imagery techniques used to contain and manage PTSD symptoms)	.50	.92
7. I use grounding techniques when I need to prevent myself from going numb, zoning out, or losing time (Examples: focus on my surroundings, pay attention to my five senses, tense and relax my muscles)	.45	.92
8. If I begin to confuse the past with the present, I notice this and work to see differences between how things are now versus how they were when I was being traumatized	.54	.92
9. I am aware of my emotions and body sensations	.58	.92
10. I am able to feel my emotions without getting overwhelmed	.60	.92
11. I am aware of, able to think about, and can control my impulses (Example: I can feel angry or depressed without doing something unhealthy)	.52	.92
12. I reach out to treatment providers if I have difficulty controlling severe unhealthy impulses despite using recovery-focused coping skills (e.g. grounding, past vs. present, containment)	.41	.92
13. I know that the traumas that I experienced were not my fault	.59	.92
14. I manage everyday life well (Examples: I regularly eat, bathe, pay bills on time, etc.)	.58	.92
15. I am able to account for all that I do that is, I don’t ‘lose time’ or find evidence of having done something I do not remember	.55	.92
16. I am able to deal with stressful situations without dissociating	.57	.92
17. I am able to maintain healthy personal and professional relationships	.57	.92
18. I value my physical well-being, and do not do things that hurt my body (Examples: I don’t cut or burn my body or attempt suicide)	.53	.92
19. I value my health and do not do things that put me at risk (Examples: I do not abuse drugs, throw up after eating, drive unsafely, have unsafe sex, etc.)	.48	.92
20. I am able to experience sadness and grieve the losses related to trauma	.62	.92
21. Life feels meaningful and rewarding	.58	.92
22. I have a generally positive view of myself	.64	.92
23. I have a generally positive view of other people	.37	.92
24. My sense of myself includes many important things beyond having been traumatized	.57	.92
25. I am able to experience sexual intimacy without intense shame, flashbacks, or dissociation, and with some pleasure	.27	.92
26. I can explore the meaning and impact related to the traumas I experienced, I can feel and express the emotions related to these traumas	.59	.92
27. All parts of myself know that we are part of the same person and that we share one body	.42	.92
28. All parts of myself are oriented to the present (know what day, month, and year it is)	.47	.92
29. I pay attention to and am curious about what different parts of myself are feeling	.58	.92
30. I’m aware of which parts of myself are contributing to my actions	.50	.92
31. All parts of myself know and can independently use recovery-focused coping skills (e.g. grounding, past vs. present, containment)	.42	.92
32. All parts of myself communicate and cooperate well	.53	.92

Items 3, 4, 12, 28, and 31 reflect capacity targets introduced in the PITQ-p.

## 1.2. Development of the PITQ-p and PITQ-t

### 1.2.1. PITQ-t development

The therapist-completed PITQ-t is a modestly updated version of the original PITQ. To explore use of the PITQ-t with patients that do not report DSS, we reorganized the measure such that DSS-related items are presented last, preceded by instructions to only rate DSS functioning if a patient experiences DSS. In response to suggestions that we clarify three PITQ items related to patient difficulties, we re-phrased and reverse-scored these items (Table 5, items 1–3). The remaining 26 items, response options, and targeted time frame are unchanged from the original PITQ.

### 1.2.2. PITQ-p development

Recent research underscores the value of querying patients’ views about their treatment. A meta-analysis of six studies comprising over 6000 treatments (Shimokawa, Lambert, & Smart, 2010) found that regularly assessing and reviewing patient treatment progress feedback with the OQ patient feedback system (e.g. Lambert, 2015) resulted in improved outcomes for patients demonstrating difficulties in making or sustaining treatment gains. A meta-analysis of 201 therapeutic alliance studies comprising over 14,000 treatments (Horvath, Del Re, Flückiger, & Symonds, 2011) found that patient-reported alliance data were generally more strongly predictive of therapeutic outcome than therapist-reported data. These findings prompted the first and second authors to develop a patient version of the PITQ. We believed that a patient version of the PITQ could afford additional insight into patients’ therapeutic progress and inform treatment planning while also offering patients opportunities to reflect on how they are doing in relation to DD treatment targets. Informed by the therapeutic alliance meta-analysis, we also anticipated that such a measure would demonstrate a stronger relationship with variables indicative of therapeutic progress than the therapist-completed PITQ-t.

In the 32-item PITQ-p, DSS items are presented last, preceded by instructions to complete these items only if relevant. Two PITQ prompts related to fusion and integration of DSS were not included because of the potential for some patients to perceive these as suggesting DSS ‘loss’ or ‘death’ (Kluft, 1993). ‬‬‬‬‬‬‬‬‬‬‬‬‬‬‬‬‬‬‬‬‬‬‬‬‬‬‬‬‬‬‬‬‬‬‬‬‬‬‬‬‬‬‬‬‬‬‬‬‬‬‬‬‬‬‬‬‬ We rephrased the remaining 27 of the PITQ’s 29 items in patient-appropriate language, and added five items (items 3, 4, 12, 28, and 31 in Table 7) in the interest of raising patient awareness of (and tracking progress related to) these adaptive capacities. To increase the PITQ-p’s potential usefulness as a routinely-administered measure, we also changed the targeted time frame to ‘in the last week’.

## 2. Method

We examined the internal consistency and convergent validity of the PITQ-t and PITQ-p in use with DD patients at baseline in a prospective longitudinal study. We hypothesized that higher PITQ-t and PITQ-p scores would be negatively correlated with concurrent validated measures indicating difficulties with emotional dysregulation, PTSD, and dissociation symptoms. We also hypothesized that PITQ-t and PITQ-p scores would be negatively correlated with self-harm and dangerous and impulsive actions, and positively correlated with positive emotions and quality of life.

### 2.1. Participants

Participants consisted of 177 patient–therapist pairs (total *N *= 354 individuals) who consented to participate in the TOP DD Network study, an IRB-approved study examining the effectiveness of an online psychoeducational programme aimed at helping early-stage DD patients learn skills to help them regulate emotion, manage DD and PTSD symptoms, and establish safety from behaviours that put their life or health at risk, including non-suicidal self-injury, suicidality, and other dangerous behaviours. We solicited participants through flyers and announcements at trauma and dissociation-focused conferences, email invitations to trauma- and dissociation-related mailing lists, and emails to therapists who had taken part in the naturalistic TOP DD study. Therapists were asked to invite a patient to participate who met diagnostic criteria for Dissociative Identity Disorder (DID; DSM-IV-TR, DSM-5), Dissociative Disorder Not Otherwise Specified (DDNOS; DSM-IV-TR), or Other Specified Dissociative Disorder (OSDD; DSM-5); had been in treatment with them for at least three months; was 18 years or older; had reliable high-speed internet access; was able to read English at the eighth grade level; could tolerate non-detailed references to trauma, safety struggles, dissociation, and brief mentions of ‘parts of self’, even if this term did not apply to them; and was willing to do approximately 2½ hours of study-related activities per week. No patients were excluded on the basis of co-morbid disorders, self-harm, or suicidality.

For the purposes of the present study, which analyses baseline data gathered prior to participants’ engagement with the Network psychoeducational programme, we only included participant pairs that unanimously indicated that the patient had DSS. See Tables 2 and 3 for therapist and patient characteristics, respectively.

### 2.2. Measures

#### 2.2.1. Progress in Treatment Questionnaire, therapist version (PITQ-t)

The PITQ-t (available at TOPDDStudy.com/PITQ-t) is a therapist-completed 29-item measure of dissociative patients’ ability to manage their emotions, symptoms, relationships, safety, and well-being. The instrument prompts therapists to assess the percentage of time their patients demonstrate expert-identified DD treatment-related behaviours over the prior six months, and includes six items completed only for patients with DSS (items 24–29). Response options range from 0% (never) to 100% (always); the first three items are reverse-scored. Higher scores indicate better adaptive functioning.

#### 2.2.2. Progress in Treatment Questionnaire, patient version (PITQ-p)

The PITQ-p (available at TOPDDStudy.com/PITQ-p) is a patient self-report measure that assesses DD patients’ ability to manage their emotions, symptoms, relationships, safety, and well-being. The PITQ-p consists of 32 expert-identified items; items 27–32 are only completed by patients who report experiencing DSS. Patients are instructed to report what percentage of time they demonstrated a behaviour during the prior week, ranging from 0% (never) to 100% (always). Higher scores indicate better adaptive functioning.

#### 2.2.3. Difficulties in emotion regulation scale (DERS; Gratz & Roemer, 2004)

The DERS is a 36-item self-report measure that assesses six domains associated with emotion regulation: emotional acceptance; emotional awareness; ability to identify and understand emotions; access to effective self-regulation strategies; ability to control impulses; and ability to engage in goal directed behaviour when upset. Response options range from 1 (almost never; 0–10%) to 5 (almost always; 91–100%); higher scores indicate greater difficulty with emotion dysregulation and poorer psychological functioning. The DERS has high internal consistency (α = .93), good overall test-retest reliability (ρ*I *= .88, *p *< .01) and adequate subscale test-retest reliability (ρ*I*s ranging from .68 to .89, all *p*s < .01) over 4–8-weeks, and adequate construct and predictive validity (Gratz & Roemer, 2004). Cronbach’s alpha was .84 in the current sample.

#### 2.2.4. Dissociative experiences scale II (DES II; Bernstein & Putnam, 1986)

The DES is a 28-item self-report measure of dissociation-related symptoms. Responses range from 0% (never) to 100% (always). Higher average scores indicate greater dissociation. A meta-analysis (van IJzendoorn & Schuengel, 1996) revealed that the measure has demonstrated high internal consistency (α = 0.93; 16 studies) and test-retest reliability (ranging from 0.78 to 0.93; six studies), as well as strong convergent validity (*r *= 0.67; 26 studies). Cronbach’s alpha was .96 in this sample.

#### 2.2.5. Posttraumatic stress checklist – civilian (PCL-C; Weathers, Litz, Huska, & Keane, 1994)

The PCL-C is a 17-item self-report measure that assesses DSM-IV-TR PTSD symptoms (APA, 2000). Patients rate the frequency of experiencing each symptom over the past month from 1 (not at all) to 5 (extremely). Higher scores indicate greater PTSD symptoms (Weathers & Ford, 1996). The measure has high overall diagnostic efficiency (0.90; Blanchard, Jones-Alexander, Buckely, & Forneris, 1996) and strong test-rest reliability (.96 with 2–3 day interval; Weathers et al., 1994). In this sample, Cronbach’s alpha was .88.

#### 2.2.6. World Health Organization Quality of Life – BREF (WHOQOL-BREF; Skevington, Lotfy, & O’Connell, 2004): psychological and social relationships domains

The WHOQOL-BREF is a 26-item measure consisting of a subset of items from the WHOQOL-100 that assesses quality of life in four domains; this study focused on the psychological and social domains. Individuals rate their quality-of-life using 5-point Likert scale response options. Responses are transformed to a scale ranging from 0–100 to enable comparisons across domains; higher domain scores indicate higher quality of life. The WHOQOL-BREF has demonstrated significant discriminant validity in the majority of the countries studied, as well as acceptable internal consistency (Cronbach’s alpha >.7) with respect to the psychological (.81) domain, and borderline internal consistency (.68) with respect to the social domain (Skevington, Lotfy, & O’Connell, 2004). In this study, Cronbach’s alpha was .79 for the psychological domain and .57 for the social domain. See Table 4 for descriptive statistics.

#### 2.2.7. Other functioning-related variables

Data indicating self-harm, dangerous actions, and impulsive actions, as well as positive emotions, were collected and analysed; see Table 4 for items’ wording and descriptive statistics.

### 2.3. Procedure

The study was approved by the Institutional Review Board at Towson University. Survey data were collected using secured, password-protected websites. All participants completed informed consent forms prior to participating. Participants were not compensated for participation.

#### 2.3.1. Analysis strategy

All analyses were performed on baseline data (i.e. data collected prior to participants engaging with the Network psychoeducational programme). To evaluate PITQ-t and PITQ-p internal consistency, we calculated Cronbach’s alphas and examined item-total statistics to assess whether internal reliability would be meaningfully improved by item deletion. To examine convergent validity, we assessed correlations between the PITQ-t and PITQ-p and the DERS, DES II, and PCL-C, as well as with patient reported self-harm, dangerous and impulsive actions, positive emotions, and psychological and social quality of life. Since higher scores for the DERS, DES II, and PCL-C are associated with greater psychological difficulty (in contrast to the PITQ-t and PITQ-p), we hypothesized that we would find negative correlations between concurrent administrations of these measures and the PITQ-t and PITQ-p. We also hypothesized that PITQ-t and PITQ-p scores would be negatively correlated with patient self-harm and dangerous and impulsive actions, and positively related to positive emotions and psychological and social quality of life. Informed by Hittner, May, and Silver (2003), we used Williams’ (1959) standard *t* to assess the significance of differences between the PITQ-t and PITQ-p’s correlations with other measures and variables. Analyses were performed in SPSS version 23; William’s standard *t* was calculated using syntax developed by Weaver and Wuensch (2013).

## 3. Results

### 3.1. PITQ-t

#### 3.1.1. Internal consistency

Cronbach’s alpha (.91) indicated that the PITQ-t demonstrated good internal consistency. Corrected item-total correlations (see Table 5) generally ranged from .33 to .69, with three exceptions: Item 2, ‘Engages in potentially self-damaging acts such as abusing substances, purging, shoplifting, driving unsafely’ (reverse scored) demonstrated a corrected item-total correlation of .24; Item 3, ‘Identity is strongly tied to being a victim of abuse’ (reverse scored) demonstrated a corrected item-total correlation of .09; and Item 22, ‘Able to experience sexual intimacy without difficulties such as intense shame, flashbacks or dissociation and with some pleasure’ demonstrated a corrected item-total correlation of .23. Examination of item-total statistics indicated that Cronbach’s alpha would be unchanged by deleting items 2 or 22, and would become .92 if Item 3 were deleted. Given the clinical salience of these items for DD patients, and that their deletion would have little impact on internal consistency, we retained them.

#### 3.1.2. Convergent validity

Correlations between the PITQ-t and the DERS, DES II, and PCL-C were significant at the .01 level (2-tailed) in the hypothesized negative direction: higher scores on the PITQ-t were associated with lower scores on the DERS (*r *= -.35), DES II (*r *= -.29), and PCL-C (*r *= -.41) (see Table 6). Correlations between the PITQ-t and unsafe behaviour in the last 30 days were also significant (*p < *.01) in the expected negative direction: self-harm (*r *= -.37), dangerous behaviour (*r *= -.31), and impulsive behaviours (*r *= -.23). Finally, the PITQ-t demonstrated the expected positive correlations (*p < *.01) with psychological (*r *= .45) and social (*r *= .22) quality of life, and positive emotions (*r *= .21) (see Table 6).

### 3.2. PITQ-p

#### 3.2.1. Internal consistency

The PITQ-p also demonstrated good internal consistency (α = .92). Corrected item-total correlations (see Table 7) generally ranged from .36 to .64, with two exceptions: Item 1 (‘I have been diagnosed with a dissociative disorder and agree that this diagnosis is correct’) demonstrated a corrected item-total correlation of .17 and Item 25 (‘I am able to experience sexual intimacy without intense shame, flashbacks, or dissociation, and with some pleasure’) demonstrated a corrected item-total correlation of .27. Examination of item-total statistics for these items indicated that reliability would not be improved by deleting either item.

#### 3.2.2. Convergent validity

As predicted, higher scores on the PITQ-p were associated with lower scores on the DERS (*r *= -.67), DES II (*r *= -.42), and PCL-C (*r *= -.47), self-harm (*r *= -.34), self-danger (*r *= -.20), and impulsive actions (*r *= -.21) (see Table 6; *p* < .01 level, 2-tailed). As hypothesized, the PITQ-p was positively correlated (*p* < .01) with psychological (*r *= .64) and social (*r *= .28) quality of life, as well as positive emotions (*r *= .31).

### 3.3. Comparative strength of relationships between PITQ-t and PITQ-p and outcomes

Results of Williams’ standard *t* (see Table 6) indicated that the differences between the PITQ-t and PITQ-p’s correlations with outcomes were significant only in relation to the DERS (*t*_174_ = 5.51, *p* < .001) and WHOQOL-BREF Psychological Domain (*t*_174_ = −3.21, *p* < .001). The PITQ-p demonstrated evidence of significantly stronger correlations than the PITQ-t with each of these measures.

## 4. Discussion

Treatment outcome research for DD patients is scarce. The lack of a reliable, validated measure for assessing progress towards resolving the myriad difficulties faced by DD patients contributes to the paucity of treatment research. To address this gap, we developed therapist (PITQ-t) and patient (PITQ-p) measures of the adaptive capacities expected to develop in DD patients over the course of trauma- and dissociation-focused treatment and examined their internal consistency and construct validity.

The PITQ-t and PITQ-p demonstrated evidence of good internal consistency, and significant, generally moderate concurrent relationships with established measures of adaptive emotion-related functioning, PTSD symptoms, dissociation, and psychological and social quality of life. Higher PITQ-t and PITQ-p scores were also related to lower levels of self-harm, dangerous behaviours, and impulsivity, as well as higher positive emotions. This suggests that higher scores on the PITQ-t and PITQ-p are associated with better functioning in each of these domains, lending support to the use of the PITQ-t and PITQ-p in evaluating DD patients’ ability to manage their emotions, symptoms, relationships, safety, and well-being.

Consistent with our rationale for developing the PITQ-p, we found that the patient-completed measure demonstrated stronger relationships with established symptom measures than the therapist-completed PITQ-t. Specifically, the correlations between the PITQ-p and measures of dissociation, PTSD, and emotion dysregulation were greater than those between these measures and the PITQ-t. The PITQ-p also demonstrated statistically stronger relationships with measures of emotional dysregulation and psychological quality of life than the PITQ-t. These findings are consistent with research indicating a stronger relationship between patients’ reports (vs. therapist reports) of alliance and treatment outcome (Horvath et al., 2011), and underscore the salience of querying patients’ perceptions about their progress in treatment.

Chronic, complex DD have been conceptualized as disorders of affect regulation related to trauma and attachment difficulties (Brand & Lanius, 2014), and difficulties in affect regulation and posttraumatic stress have been found to predict increased dissociation (Briere, Hodges, & Godbout, 2010). Our findings add to the literature indicating the importance of assessing these areas of functioning in DD patients. The PITQ-p and PITQ-t assess adaptive capacities related to these difficulties, and the TOP DD naturalistic study demonstrated that these capacities develop over time in DD treatment (Brand, Classen, Lanius et al., 2009, 2013).

### 4.1. Limitations and areas for future study

This self-selected sample population consisted predominantly of female Caucasian patients that presented with DSS seeing primarily female therapists. Future studies should strive to increase representation of groups under-represented here, examine the validity and utility of the measures with patients who do not demonstrate or endorse DSS, and further examine the PITQ-t and PITQ-p’s consistency and validity in terms of replication and extension. This includes examining the measures’ temporal stability, discriminant validity, and utility with DD patients as well as with other populations that experience dissociation, including patients with the dissociative subtype of PTSD (DSM-5; Lanius, Brand, Vermetten, Frewen, & Spiegel, 2012; Stein et al., 2013; Wolf et al., 2012) and/or those reporting complex trauma histories (Cook et al., 2005; Courtois, 2004; Ford & Courtois, 2009). Given that the measures studied in relation to the patient self-report version of the PITQ were each self-report measures, it is possible that shared method variance may be meaningfully contributing to the PITQ-p’s stronger relationships with these measures. Future studies could include additional observational and/or collateral outcome measures as well as measures of divergent constructs and examine this question through the multi-trait, multi-method matrix approach (Campbell & Fiske, 1959). The measures’ relationships with other instruments and variables of interest should also be examined over multiple time-points within a longitudinal study, a process that is currently underway. ‬‬‬‬‬‬‬‬‬Future studies could also examine if completing the PITQ-p during the course of therapy helps patients acquire awareness of the links between adaptive abilities and improved psychological quality of life more rapidly than those that do not complete the measure, and could examine whether regular use of the PITQ-p improves therapeutic progress and/or outcomes. Finally, in the present study, patients completed single-item variables that assessed self-harm and impulsive and dangerous behaviours over the prior 30 days. Future studies should match the time frame for patient-reported outcomes with the seven-day time frame used by the PITQ-p.

### 4.2. Conclusions

Our findings suggest that the PITQ-t and PITQ-p are promising measures of adaptive functioning in DD patients and merit use and additional research in relation to the assessment of therapeutic progress with patients with DD. These measures seem to capture important aspects of DD patients’ development of adaptive capacities in managing safety, emotion, symptoms, and relationships, and offer researchers and clinicians a means of tracking progress and studying the process of therapeutic change with this under-studied population. They are also promising tools for evaluating patients’ relative strengths and areas of difficulty, information that can guide treatment planning (Lambert, Gregersen, & Burlingame, 2004; Pinsof & Chambers, 2009) and facilitate treatment responsiveness (Stiles, Honos-Webb, & Surko, 1998).

Therapists have informed us that these measures have been useful in treatment planning and in discussing treatment progress with patients. The PITQ-p may be useful in facilitating treatment responsiveness by helping clinicians efficiently evaluate multiple areas of their DD patients’ functioning. Asking patients to periodically complete a PITQ-p enables therapists to rapidly assess the relative presence or absence of a range of expert-identified abilities to be targeted in treatment, allowing therapists to quickly assess areas of functioning that are frequently challenging for DD patients and providing important information that can facilitate treatment planning‬‬‬. ‬‬‬ Completing the PITQ-p also encourages patients to reflect on their progress towards treatment goals, which may, in turn, encourage increased use of the skills assessed within the measure.

## Note

1. Dissociative self-states are sometimes referred to as dissociative identities, personalities, alters, or ‘parts’.
